# Case of Scirrhus of the Pancreas

**Published:** 1851-10

**Authors:** J. R. McClurg

**Affiliations:** New Garden, Chester County, Pennsylvania


*Case of Scirrhus of the Pancreas.* By J. R. McClurg, M. D., of New Garden, Pennsylvania. Communicated by Prof. Dunglison.

Thomas J. Yeatman, blacksmith, aged 50 years, of a bilious temperament, was taken sick about Christmas of the year 1850, at which time he complained of a pain, weight or oppression in the epigastrium, which became more severe upon pressure—no appetite and yet no sickness at the stomach; a great restlessness of the system and an inability to lie in bed. The cause of this, the patient said, was the oppression in the region of the stomach.

A blister was applied over the epigastrium, which appeared to remove the pain for a day or two, but as soon as it had dried up the oppression returned. The patient was still able to walk a little. Two weeks after the first attack, he became worse, and went to bed; a physician was sent for who pronounced his disease bilious fever and treated him accordingly. At this time he complained of a continuance of all the above symptoms and of an aggravated character. He was now able to lie in bed, which for some time past he could not do;—had fever—pain in the left side over the spleen—still no sickness nor vomiting;—no disturbance of the brain or lungs;—no tenderness or pain in the right side;—the bowels were costive, requiring a large amount of medicine to produce evacuations. One week after the above attack of fever, erysipelas appeared in the face, one eye became almost closed, but after two or three days the erysipelas entirely disappeared. He was confined to his bed and room between four and five weeks, at the end of which time he became able to walk out, but was still in a very weak condition. The disease still remained in the stomach, as in the very first attack; poor appetite, no disposition to sleep. Being at this time unable to lie in bed, he sat up all night in his chair, or walked the room in order to obtain relief from the pain, which had become constant. Sometimes it would be worse in the region of the stomach, then in the left side, then perhaps in the back. He was at this time, and indeed from the first attack, unable to stand erect, but bent forward as if to relax the abdominal muscles. When the pain was most severe, the application of a warm poultice or hot salt to the seat of pain would give relief for a short time. Having been under the care of a regular physician or physicians for three months or more, and receiving but little benefit from their treatment, he visited Lancaster, and consulted a "*Dutch Doctor*," who promised to cure him; but as he grew rapidly worse in his hands, he abandoned the case, which now came under my care.

Thus on the 9th day of May, four and a half months after Mr. Yeatman was first taken sick, I saw and examined his case for the first time, and found him in the following condition. He was quite feeble, but still able to walk about the house;—his skin was dry, of a straw color; the countenance wore a wild, anxious expression, the tongue was covered with a thick yellow coat; the breath quite foul; the pulse quick, corded and ninety per minute ;—great oppression deep in the epigastric region, and remarkably tender on pressure, presenting to the eye a fulness, and to the touch a thickened or knotted condition ;—a decided pulsation was perceptible in the epigastrium, caused by the pulsation of the abdominal aorta ;—pain in the left hypochondriac region, also in the back ; tenderness on pressure over the colon, from the caput coli to the sigmoid flexure ; the bowels costive; great restlessness of the system and inability to lie in bed ; he sat up all night in his chair, and had done so for the last three or four weeks ; unable to stand erect, but bent forward; sat with his feet drawn up on the chair and his knees in contact with the thorax or chin ; poor appetite ; no sickness at the stomach ; no pain in the right side nor along the course of the spine upon pressure. Cupping and blistering appeared to have little or no effect at this time in removing the pain. I gave the blue pill, extract of taraxacum and hyoscyamus, together with the iodide of potassium, without any benefit in the case. I then prescribed an aloetic and a saline purge occasionally, together with the sulphate of quinine and carb. of iron, and after administering *many* other medicines of a tonic and antispasmodic character, I gave the nitrate of silver freely, without perceiving any benefit. The disease seemed to be advancing gradually to a fatal termination notwithstanding every effort used to arrest it. The pain was becoming more severe every day ; no appetite, or ability to walk without assistance ; pain *now in the right side*, and over the whole abdomen. His suffering became very great. When the pain was most severe, he would get into bed, with his head and shoulders elevated, grasp one thigh with each hand, and with his feet up towards the ceiling or perhaps thrown back over his head, making the small of the back a pivot, would turn round and round in bed, groaning most mournfully and writhing as if in intense agony. And thus it would continue for hours unless relieved by large doses of opium or sulphate of morphia. The medicine which seemed to have the most influence in relieving his sufferings was the following mixture *prepared for the emergency of the case alone.* ℞. Vinegar ℥vi., saturate with the carbonate of potash, then add of the sulphate of morphia, gr.xv.

a large table spoon full to be given every hour whilst the pain continued. Two or three spoonfuls generally afforded him rest for five or six hours, when it became necessary to repeat the dose, thus keeping him under the constant influence of morphia to prevent intense suffering. Three or four weeks before his death, the hands, feet and abdomen, became greatly swollen; the urine of a very dark red color, and hiccough was almost constant. He gradually sank, as if some singular and fatal disease was seated at the very fountain of life or centre of sympathy, crushing the life slowly, yet certainly, of a strong and vigorous man. Mr. Yeatman died on the 18th of August, having been sick about eight months.

*On inspection*, the disease appeared to be of a scirrho-scrofulous character. On opening the abdomen, much effused fluid of a dark, chocolate color filled the peritoneal sac, which appeared to be in a diseased condition.

The liver was somewhat enlarged, hard and tuberculated; a few of the tubercles contained pus. The gall bladder was healthy, of the natural size, and contained three hexagonal calculi, each of which was about the size of a large chestnut. The cavity of the stomach, or mucous membrane, appeared to be in a normal condition—the greater curvature free from disease, but in the smaller curvature was found a large scirrhus mass, embracing one third of the stomach, the whole of the pancreas, solar plexus, the aorta and accompanying vessels, and adhering to the diaphragm, the liver, the arch of the colon and omentum. The stomach and diseased mass, were removed together from the body, by dissecting them from the above points of adhesion, which was a difficult task. The spleen was perfectly healthy, as were also the kidneys, heart and lungs. The solar plexus being involved in the diseased mass, was the cause, no doubt, of the great, very great, suffering of the patient. And as numerous branches emanate from it, which supply the diaphragm, stomach, liver, spleen, small and large intestines, kidneys, supra-venal capsules and the spermatic vessels, we need not be surprised that the patient occasionally experienced pain in these various organs. That the stomach and pancreas, the latter in particular, was the original seat of this disease, and that the surrounding organs became subsequently involved, there is not the least shadow of doubt in my mind. The liver was implicated, no doubt, long after the above, as symptoms clearly indicate, for there never was the least pain or uneasiness felt in the right side, until about a month or two before death. The cause of the disease is very uncertain, but most likely it was produced by the patient, some two years previous to his last sickness, having carried a load of coal from the wagon into the cellar in a tub, which pressed hard against the upper part of the stomach giving him pain at the time, and ever after some uneasiness or a tired feeling in the epigastrium.

This is an interesting case on account of the peculiar symptoms developed,—there being no similar case on record, so far as I have seen—also on account of the great suffering of the patient,—the character of the pain—his position when the pain was most severe—the manner in which he sat, with his knees in contact with the thorax or chin—being unable to stand erect; the absence of sickness and vomiting, &c. and it is also interesting on account of its pathological conditions.

*New Garden, Chester County, Pennsylvania, August 19th*, 1851.

